# Erianin inhibits high glucose-induced retinal angiogenesis via blocking ERK1/2-regulated HIF-1α-VEGF/VEGFR2 signaling pathway

**DOI:** 10.1038/srep34306

**Published:** 2016-09-28

**Authors:** Zengyang Yu, Tianyu Zhang, Chenyuan Gong, Yuchen Sheng, Bin Lu, Lingyu Zhou, Lili Ji, Zhengtao Wang

**Affiliations:** 1 The Shanghai Key Laboratory of Complex Prescription, MOE Key Laboratory for Standardization of Chinese Medicines, and SATCM Key Laboratory for New Resources and Quality Evaluation of Chinese Medicines, Institute of Chinese Materia Medica, Shanghai University of Traditional Chinese Medicine, Shanghai 201203, China; 2 Center for Drug Safety Evaluation and Research, Shanghai University of Traditional Chinese Medicine, Shanghai 201203, China

## Abstract

Erianin is a natural compound found in *Dendrobium chrysotoxum* Lindl. Diabetic retinopathy (DR) is a serious and common microvascular complication of diabetes. This study aims to investigate the inhibitory mechanism of erianin on retinal neoangiogenesis and its contribution to the amelioration of DR. Erianin blocked high glucose (HG)-induced tube formation and migration in choroid-retinal endothelial RF/6A cells. Erianin inhibited HG-induced vascular endothelial growth factor (VEGF) expression, hypoxia-inducible factor 1-alpha (HIF-1α) translocation into nucleus and ERK1/2 activation in RF/6A and microglia BV-2 cells. MEK1/2 inhibitor U0126 blocked HG-induced HIF-1α and ERK1/2 activation in both above two cells. In addition, erianin abrogated VEGF-induced angiogenesis *in vitro* and *in vivo*, and also inhibited VEGF-induced activation of VEGF receptor 2 (VEGFR2) and its downstream cRaf-MEK1/2-ERK1/2 and PI3K-AKT signaling pathways in RF/6A cells. Furthermore, erianin reduced the increased retinal vessels, VEGF expression and microglia activation in streptozotocin (STZ)-induced hyperglycemic and oxygen-induced retinopathy (OIR) mice. In conclusion, our results demonstrate that erianin inhibits retinal neoangiogenesis by abrogating HG-induced VEGF expression by blocking ERK1/2-mediated HIF-1α activation in retinal endothelial and microglial cells, and further suppressing VEGF-induced activation of VEGFR2 and its downstream signals in retinal endothelial cells.

Diabetic retinopathy (DR) is one of the most serious and common complications of diabetes mellitus (Diabetes, DM)^1^. It is a chronic microvascular complication caused by hyperglycemia during the development of DM, and nearly one-third of the diabetic patients have signs of retinopathy and one-tenth may have proliferative DR (PDR) or diabetic macular edema (DME), which are two states of vision-threatening DR^2^. Vision loss or even blindness due to DR seriously affects the living quality of life in diabetic patients and causes huge health burden. However, there is still no effective therapeutic strategy or drug for DR treatment. With the rising living standards, the incidence of diabetes is increasing day by day. It is estimated that the number of people having diabetes is expected to exceed half a billion by 2030 year^3^. Moreover, a report shows that about more than 90 million diabetic patients will have DR, of which about 17 million have PDR and 21 million have DME in the developed countries^4^. Thus, as one of the most common complications of diabetes, DR may be a big disease for people to conquer in the future.

According to the progression of DR, it is generally classified into two stages: the early non-proliferative DR (NPDR) stage and the later PDR stage^5^. The hallmark of PDR is retinal neovascularization, which is a rapid and uncontrolled process of ocular angiogenesis, leading to the formation of fragile and leaky vasculature, finally causing irreversible damage to retina and even blindness without treatment^2^,^6^. Thus, anti-angiogenesis has been a promising and feasible strategy for DR treatment recently^7^. VEGF is a well-known pro-angiogenic factor for promoting neovascularization. Therefore, there are some anti-VEGF agents proved to be potentially useful for DME or PDR treatment, including bevacizumab (Avastin, Genentech), ranibizumab (Lucentis, Genentech) and aflibercept (Eylea, Regeneron), of which ranibizumab is the only approved drug for intravitreal use in DME treatment in United States and Europe^8^,^9^. Also, there is increasing evidence that inflammation plays a critical role in the pathogenesis of DR, and the elevated pro-inflammatory factors including tumor necrosis factor-α (TNF-α), nitric oxide (NO) and intracellular adhesion molecule-1 (ICAM-1) contributed to the blood-retinal barrier (BRB) breakdown at the stage of NPDR^10^,^11^. Besides, recent studies demonstrated there was a feed-forward loop between VEGF and cytosolic phospholipase A_2_ (cPLA_2_)/cyclooxygenase-2 (COX2)/prostaglandin (PG) axis, which is a well-known signaling pathway involved in inflammation^12^,^13^.

Natural product erianin (The chemical structure was shown in Supplementary Fig. 1) is the major bibenzyl compound found in the traditional Chinese medicine *Dendrobium chrysotoxum* Lindl. It is generally used as the chemical marker for the quality control of *D. chrysotoxum*, which is a species of medicinal *Dendrobium* (Shi-Hu) indexed in the Chinese Pharmacopoeia (2010 version)^14^. According to the records of ancient medical books in China, medicinal *Dendrobium* is generally used for improving eyesight in clinic. Our previous studies have already shown that *D. chrysotoxum* attenuated the development of DR *in vivo* by inhibiting retinal angiogenesis and inflammation^15^,^16^. In addition, the previous study in our lab has demonstrated that erianin blocked tumor angiogenesis, which contributed to its inhibition on tumor growth^17^. Whether erianin can also inhibit retinal neoangiogenesis during the process of DR? What’s the engaged mechanism involved in erianin-induced inhibition on retinal angiogenesis? All those above problems remain unknown. There are more and more reports about the inhibitors coming from natural products for retinal neovascularization including curcumin, isoliquiritigenin, genistein, and all those natural products may be used as the leading compound for designing more efficacious and safe drugs for the treatment of ocular neovascular diseases including DR^18^. Here, we investigated the attenuation of erianin on DR by inhibiting retinal angiogenesis *in vitro* and *in vivo*, along with the elucidation of the signaling pathways that may be involved.

## Results

### Erianin inhibited HG-induced tube formation and migration in RF/6A cells

**Figure 1:**
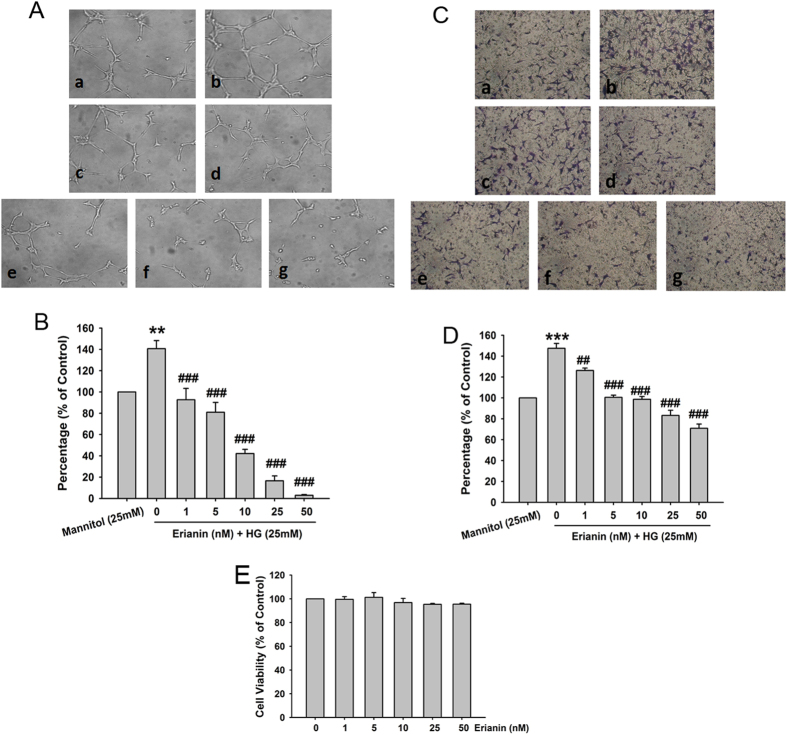
Erianin inhibited HG-induced tube formation and migration in RF/6A cells. (**A**) Cells were pretreated with different concentrations of erianin (0–50 nM) for 15 min, and then incubated with HG (25 mM) for 4 h. The formed tube-like structures were taken under a microscope. (**B**) The tube-like structures were quantified by manual counting, and presented as percentage of control (n = 3). (**C**) Cells were pretreated with various concentrations of erianin (0–50 nM) for 15 min, and then incubated with HG (25 mM) for 8 h. The non-migrated cells were wiped off with a cotton swab, and the migrated cells were stained by crystal violet. Images were taken under a microscope. (**D**) The migrated cells were quantified by manual counting, and presented as percentage of control (n = 3). a Control, b HG (25 mM), c HG + erianin (1 nM), d HG + erianin (5 nM), e HG + erianin (10 nM), f HG + erianin (25 nM), g HG + erianin (50 nM). (**E**) RF/6A cells were incubated with different concentrations of erianin (0–50 nM) for 48 h. Cell viability were determined by MTT assay (n = 5). Data = Means ± SEM. **P < 0.01, ***P < 0.001 vs. control; ^*##*^*P* < 0.01, ^*###*^*P* < 0.001 vs. HG.

Hyperglycemia produced during the development of diabetes will cause damage and promote angiogenesis in retinal endothelial cells^19^,^20^. Firstly, we observed the effects of erianin on high glucose (HG)-induced angiogenesis in retinal endothelial cells, and the monkey choroidal retinal vascular endothelial (RF/6A) cell line was used in this study. As compared with osmotic mannitol (25 mM) control group, the tube formation (Fig. 1A,B) and cell migration (Fig. 1C,D) were both enhanced in HG (25 mM)-treated RF/6A cells. However, erianin abrogated HG-induced both tube formation (Fig. 1A,B) and cell migration (Fig. 1C,D) in RF/6A cells in the concentration-dependent manner. In addition, erianin (1–50 nM) alone had no obvious cytotoxicity on RF/6A cells after incubated with cells for 48 h (Fig. 1E). The results imply that the inhibition of erianin on HG-induced tube formation and migration in RF/6A cells was not due to its cytotoxicity. We also observed the inhibitory effect of bevacizumab (Avastin) on HG-induced RF/6A cell tube formation *in vitro*. The results (Supplementary Fig. 2) showed that bevacizumab (0.5–10 mg/ml) inhibited HG-induced tube formation in RF/6A cells in the concentration-dependent manner, and the inhibition of erianin (50 nM) was better than 5 mg/ml bevacizumab, but weaker than 10 mg/ml bevacizumab. The molecular weight of bevacizumab is about 149 KDa. After conversion, 5 mg/ml bevacizumab is about 33.6 μM bevacizumab. So, we can see that the inhibition of erianin on HG-induced tube formation *in vitro* is better than bevacizumab.

### Erianin inhibited HG-induced HIF-1α-mediated VEGF expression in RF/6A cells

**Figure 2:**
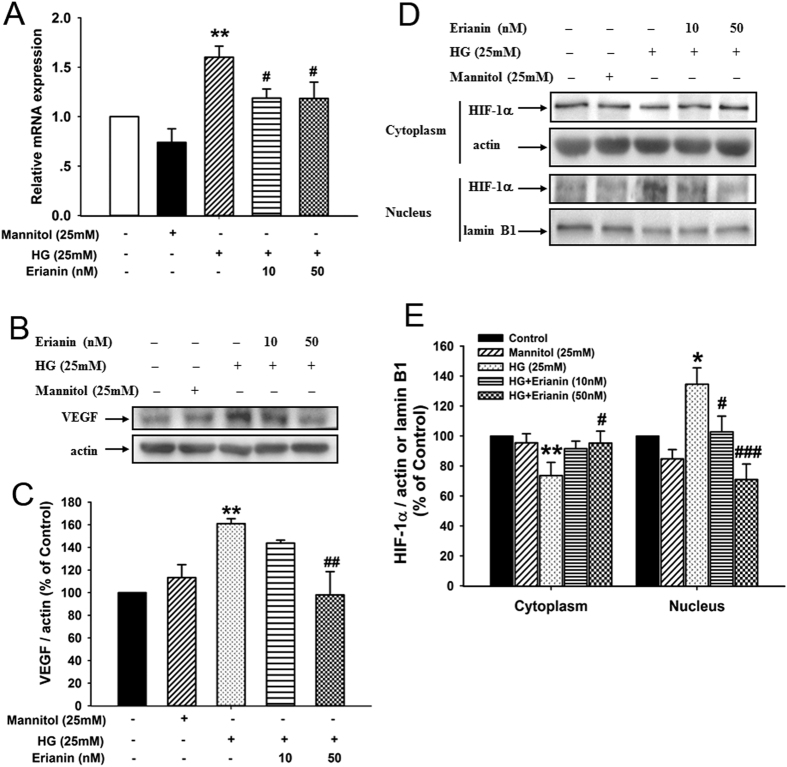
Erianin inhibited HG-induced HIF-1α-mediated VEGF expression in RF/6A cells. (**A**) Erianin reduced the HG-induced increased VEGF mRNA expression (n = 5). (**B**) Erianin reduced the HG-induced increased VEGF protein expression. Representative blots for VEGF and actin, and the results represent three independent experiments. (**C**) The quantitative densitometric analysis of VEGF and actin, and the results were presented as percentage of control (n = 3). (**D**) Erianin abrogated HG-induced HIF-1α translocation from cytoplasm into nucleus. Representative blots for HIF-1α in cytoplasm or nucleus, and the results represent six independent experiments. (**E**) The quantitative densitometric analysis of HIF-1α, lamin B1, and actin, and the results were presented as percentage of control (n = 6). Data = Means ± SEM. *P < 0.05, **P < 0.01 vs. control; ^*#*^*P* < 0.05, ^*##*^*P* < 0.01, ^*###*^*P* < 0.001 vs. HG.

As shown in Fig. 2A, HG (25 mM) increased the mRNA expression of VEGF in RF/6A cells, however erianin (10, 50 nM) reduced the HG-induced increased VEGF mRNA expression. Erianin (50 nM) also decreased the HG-induced increased protein expression of VEGF in RF/6A cells (Fig. 2B,C). Next, the translocation of HIF-1α from cytoplasm into nucleus was observed. The results showed that HG (25 mM) decreased HIF-1α expression in cytoplasm, but increased its expression in nucleus, indicating that HG induced the transcriptional activation of HIF-1α (Fig. 2D,E). However, after erianin (10, 50 nM) treatment, HG-induced HIF-1α translocation into nucleus was reduced (Fig. 2D,E).

### Erianin inhibited HG-induced HIF-1α-mediated VEGF expression in BV-2 cells

**Figure 3:**
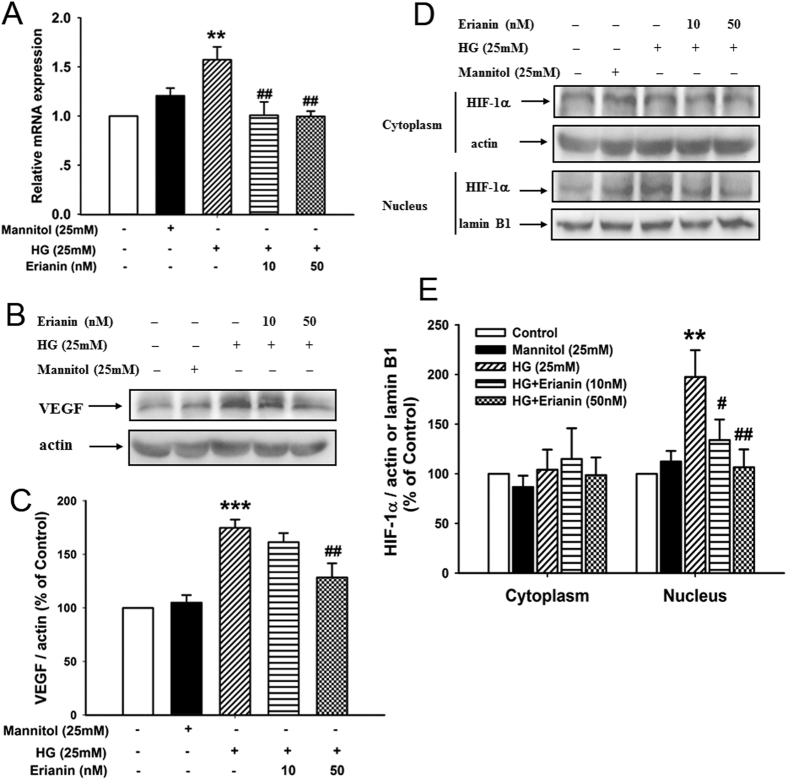
Erianin inhibited HG-induced HIF-1α-mediated VEGF expression in BV-2 cells. (**A**) Erianin reduced the HG-induced increased VEGF mRNA expression (n = 4). (**B**) Erianin reduced the HG-induced increased VEGF protein expression. Representative blots for VEGF and actin, and the results represent four independent experiments. (**C**) The quantitative densitometric analysis of VEGF and actin, and the results were presented as percentage of control (n = 4). (**D**) Erianin abrogated HG-induced HIF-1α translocation from cytoplasm into nucleus. Representative blots for HIF-1α in cytoplasm or nucleus, and the results represent five independent experiments. (**E**) The quantitative densitometric analysis of HIF-1α, lanmin B1, and actin, and the results were presented as percentage of control (n = 5). Data = Means ± SEM. **P < 0.01, ***P < 0.001 vs. control; ^*#*^*P* < 0.05, ^*##*^*P* < 0.01 vs. HG.

As shown in Fig. 3A, erianin (10, 50 nM) reduced the increased VEGF mRNA expression induced by HG (25 mM) in microglia BV-2 cells. Moreover, erianin (50 nM) also decreased the HG-induced increased VEGF protein expression in BV-2 cells (Fig. 3B,C). Next, the results of the translocation of HIF-1α into nucleus showed that erianin (10, 50 nM) abrogated HG (25 mM)-induced HIF-1α transcriptional activation in BV-2 cells (Fig. 3D,E). In addition, erianin (1–50 nM) alone had no obvious cytotoxicity on BV-2 cells (Supplementary Fig. 3). The results imply that the inhibition of erianin on HG-induced HIF-1α nuclear translocation and VEGF expression in BV-2 cells was not due to its cytotoxicity.

### Erianin inhibited ERK1/2-mediated HIF-1α activation in RF/6A and BV-2 cells

**Figure 4:**
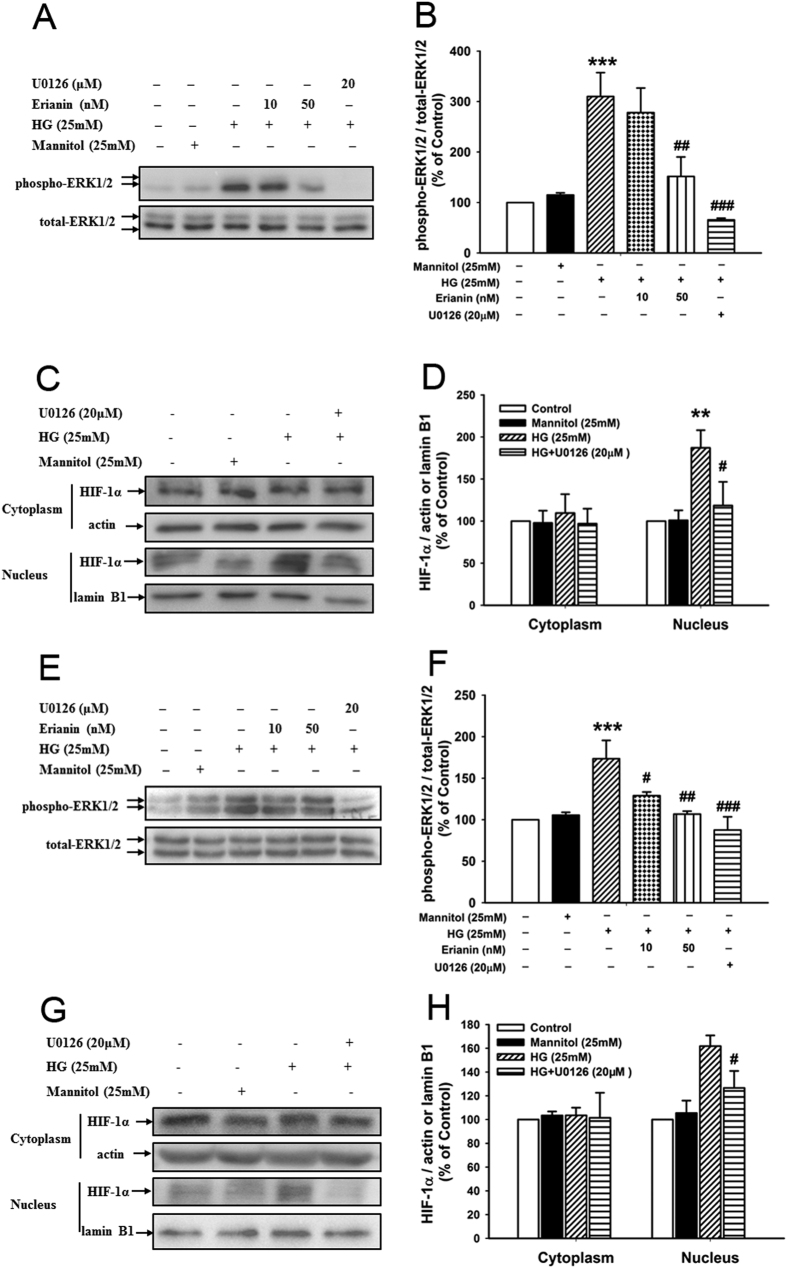
Erianin inhibited ERK1/2-mediated HIF-1α activation in both RF/6A and BV-2 cells. (**A**) Erianin inhibited HG-induced ERK1/2 activation in RF/6A cells. Representative blots for phospho-ERK1/2 and ERK1/2, and the results represent four independent experiments. (**B**) The quantitative densitometric analysis of phospho-ERK1/2 and ERK1/2, and the results were presented as percentage of control (n = 4). (**C**) U0126 inhibited HG-induced HIF-1α translocation from cytoplasm into nucleus in RF/6A cells. Representative blots for HIF-1α in cytoplasm or nucleus, and the results represent five independent experiments. (**D**) The quantitative densitometric analysis of HIF-1α, lanmin B1, and actin, and the results were presented as percentage of control (n = 5). (**E**) Erianin inhibited HG-induced ERK1/2 activation in BV-2 cells. Representative blots for phospho-ERK1/2 and ERK1/2, and the results represent five independent experiments. (**F**) The quantitative densitometric analysis of phospho-ERK1/2 and ERK1/2, and the results were presented as percentage of control (n = 5). (**G**) U0126 inhibited HG-induced HIF-1α translocation from cytoplasm into nucleus in BV-2 cells. Representative blots for HIF-1α in cytoplasm or nucleus, and the results represent five independent experiments. (**H**) The quantitative densitometric analysis of HIF-1α, lamin B1, and actin, and the results were presented as percentage of control (n = 5). Data = Means ± SEM. **P < 0.01, ***P < 0.001 vs. control; ^*#*^*P* < 0.05, ^*##*^*P* < 0.01, ^*###*^*P* < 0.001 vs. HG.

As shown in Fig. 4A,B, the results showed that erianin (50 nM) blocked HG (25 mM)-induced ERK1/2 phosphorylation in RF/6A cells, just like the inhibition induced by the MEK1/2 inhibitor U0126. As pointed out in previous Fig. 2D,E, erianin inhibited HG (25 mM)-induced HIF-1α translocation from cytoplasm into nucleus in RF/6A cells. Similarly, U0126 had the same function (Fig. 4C,D).

Next, the results in microglia BV-2 cells (Fig. 4E,F) showed that erianin (10, 50 nM) also abrogated HG (25 mM)-induced ERK1/2 phosphorylation in BV-2 cells, just like the inhibition induced by U0126. In addition, just the same as erianin (Fig. 3D,E), U0126 also blocked HG (25 mM)-induced HIF-1α translocation from cytoplasm into nucleus in BV-2 cells (Fig. 4G,H).

### Erianin abrogated VEGF-induced angiogenic responses in RF/6A cells and in matrigels *in vivo*

**Figure 5:**
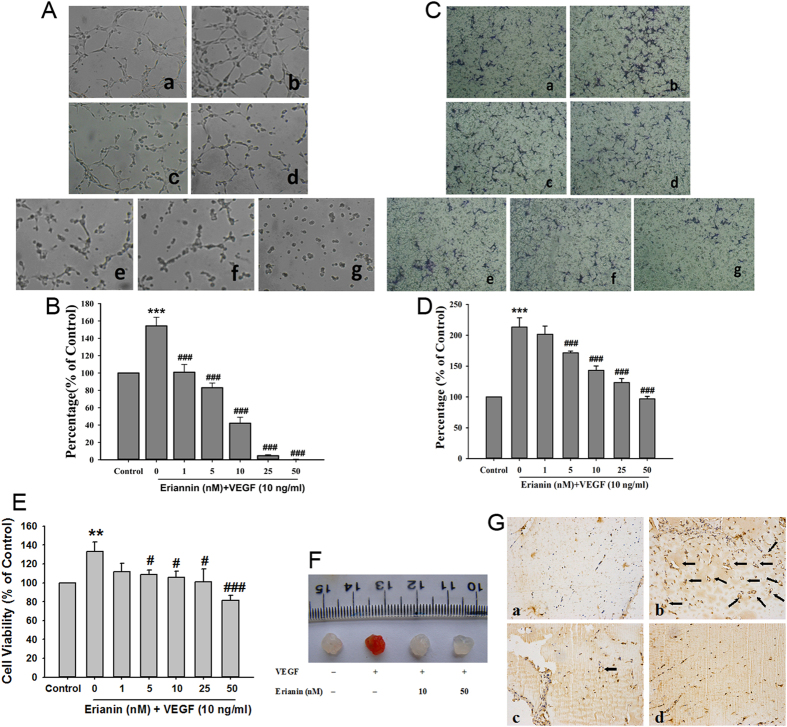
Erianin abrogated VEGF-induced angiogenesis in RF/6A cells. (**A**) Cells were pretreated with different concentrations of erianin (0–50 nM) for 15 min, and then incubated with VEGF (10 ng/ml) for 4 h. The formed tube-like structures were taken under a microscope. (**B**) The tube-like structures were quantified by manual counting, and presented as percentage of control (n = 3). (**C**) Cells were pretreated with various concentrations of erianin (0–50 nM) for 15 min, and then incubated with VEGF (10 ng/ml) for 8 h. The non-migrated cells were wiped off with a cotton swab, and the migrated cells were stained by crystal violet. Images were taken under a microscope. (**D**) The migrated cells were quantified by manual counting, and presented as percentage of control (n = 3). a Control, b VEGF (10 ng/ml), c VEGF + erianin (1 nM), d VEGF + erianin (5 nM), e VEGF + erianin (10 nM), f VEGF + erianin (25 nM), g VEGF + erianin (50 nM). (**E**) RF/6A cells were pretreated with different concentrations of erianin (0–50 nM) for 15 min, and then incubated with VEGF (10 ng/ml) for another 48 h. Cell viability were determined by MTT assay (n = 4). Data = Means ± SEM. **P < 0.01, ***P < 0.001 vs. control; ^*#*^*P* < 0.05, ^*###*^*P* < 0.001 vs. VEGF. (**F**) Matrigels containing VEGF (10 ng/ml) and erianin (10, 50 nM) was injected into mice. After 7 days, the mice were sacrificed and the matrigels were taken out and pictured. (**G**) The 5 μm sections of matrigels were stained with CD31 and photographed. a Control, b VEGF, c VEGF + erianin (10 nM), d VEGF + erianin (50 nM).

The formation of elongated and robust tube-like structures induced by VEGF (10 ng/ml) in RF/6A cells was abrogated by erianin in the concentration-dependent manner (Fig. 5A,B). We also observed the inhibitory effect of bevacizumab on VEGF-induced RF/6A cell tube formation *in vitro*. The results (Supplementary Fig. 4) showed that bevacizumab (0.5–10 mg/ml) reduced VEGF-induced tube formation in RF/6A cells in the concentration-dependent manner, and the inhibition of erianin (50 nM) was better than 5 mg/ml bevacizumab, but weaker than 10 mg/ml bevacizumab. So, the inhibition of erianin on VEGF-induced tube formation *in vitro* is also better than bevacizumab. In addition, erianin (5–50 nM) also inhibited the VEGF-induced migration of RF/6A cells (Fig. 5C,D). As shown in Fig. 5E, erianin (5–50 nM) inhibited the increased cell viability induced by VEGF (10 ng/ml) in the concentration-dependent manner. In addition, the anti-angiogenic activity of erianin *in vivo* was observed in VEGF-treated matrigels in mice. As compared with control, VEGF-treated matrigels appeared red color, and the number of CD31 staining vessels was increased, indicating the formation of a functional vasculature inside the matrigels (Fig. 5F,G). However, with erianin (10, 50 nM) treatment, the red color in matrigels become diminished and CD31 staining vessels was decreased (Fig. 5F,G).

### Erianin blocked VEGF-induced activation of VEGFR2 and its downstream cRaf-MEK1/2-ERK1/2 and PI3K-AKT signaling cascades in RF/6A cells

**Figure 6:**
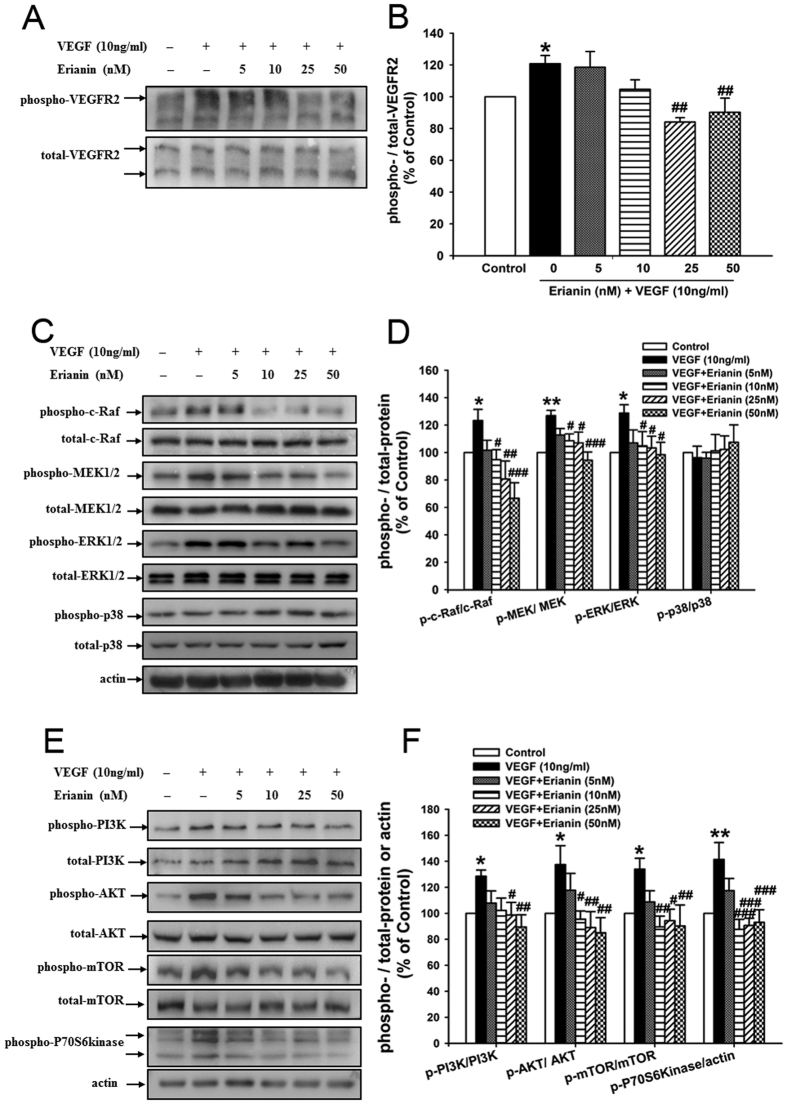
Erianin blocked VEGF-induced activation of VEGFR2 and its downstream cRaf-MEK1/2-ERK1/2 and PI3K-AKT signaling cascades in RF/6A cells. (**A**) Erianin inhibited VEGF-induced VEGFR2 phosphorylation. Representative blots for phospho-VEGFR2 and VEGFR2, and the results represent three independent experiments. (**B**) The quantitative densitometric analysis of phospho-VEGFR2 and VEGFR2, and the results were presented as percentage of control (n = 3). (**C**) Erianin inhibited VEGF-induced activation of cRaf-MEK1/2-ERK1/2 signaling cascade. Representative blots for phospho-cRaf, phospho-MEK1/2, phospho-ERK1/2, phospho-p38, cRaf, MEK1/2, ERK1/2, and p38, and the results represent at least three independent experiments. (**D**) The quantitative densitometric analysis of phospho-cRaf, phospho-MEK1/2, phospho-ERK1/2, phospho-p38, cRaf, MEK1/2, ERK1/2, and p38, and the results were presented as percentage of control (n = 3–6). (**E**) Erianin inhibited VEGF-induced activation of PI3K-AKT signaling cascade. Representative blots for phospho-PI3K, phospho-AKT, phospho-mTOR, phospho-P70S6kinase, PI3K, AKT, mTOR, and actin, and the results represent at least three independent experiments. (**F**) The quantitative densitometric analysis of phospho-PI3K, phospho-AKT, phospho-mTOR, phospho-P70S6kinase, PI3K, AKT, mTOR, and actin, and the results were presented as percentage of control (n = 3–6). Data = Means ± SEM. *P < 0.05, **P < 0.01 vs. control; ^*#*^*P* < 0.05, ^*##*^*P* < 0.01, ^*###*^*P* < 0.001 vs. VEGF.

As shown in Fig. 6A,B, Erianin (25, 50 nM) abrogated VEGF-induced phosphorylated activation of VEGFR2 in RF/6A cells. Erianin (10, 25, 50 nM) inhibited VEGF-induced ERK1/2 phosphorylation but had no effect on VEGF-induced phosphorylation of p38 (Fig. 6C,D). In addition, erianin (10, 25, 50 nM) also blocked VEGF-induced phosphorylated activation of c-Raf and MEK1/2, which are both upstream kinases of ERK1/2 (Fig. 6C,D). Furthermore, erianin (25, 50 nM) abrogated VEGF-induced phosphorylation of PI3K in RF/6A cells (Fig. 6E,F). Erianin (10, 25, 50 nM) also blocked VEGF-induced phosphorylation of AKT, mTOR and P70S6kinase, which are all downstream kinases of PI3K (Fig. 6E,F).

### Erianin abrogated retinal neovascularization in STZ-induced hyperglycemic mice

**Figure 7:**
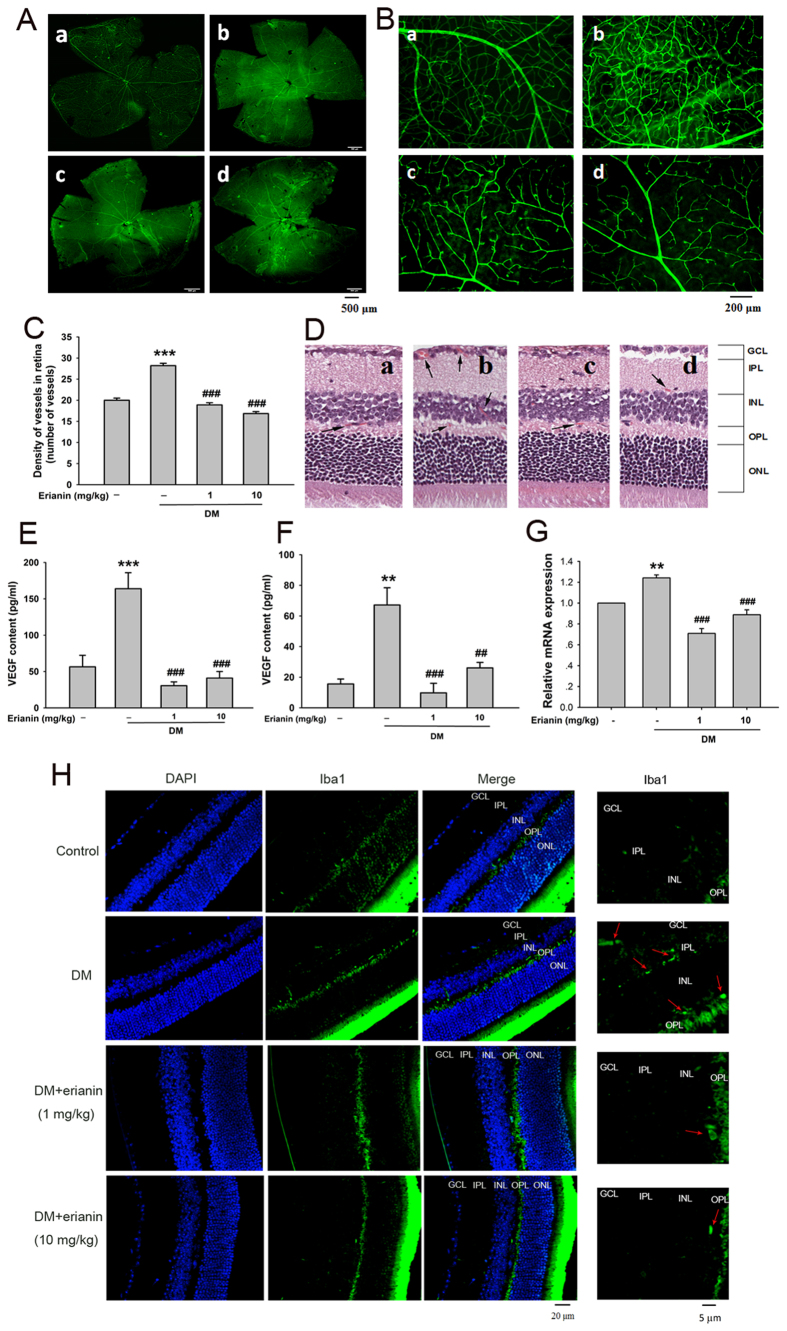
Erianin ameliorated DR in STZ-induced hyperglycemic mice. (**A**) The whole mounting of CD31-stained retinas. (**B**) The partial enlarged picture of CD31-stained retinas. (**C**) Quantitative results of CD31-stained retinal vessels (n = 6). (**D**) Representative pictures of H&E staining of retinas. a Control group, b DM group, c DM + erianin (1 mg/kg) group, d DM + erianin (10 mg/kg) group. (**E**) VEGF content in serum (n = 7–9). (**F**) VEGF content in vitreous cavity (n = 7–9). (**G**) The mRNA expression of retinal VEGF (n = 7–9). Data = Means ± SEM. **P < 0.01, ***P < 0.001 vs. control; ^*##*^*P* < 0.01, ^*###*^*P* < 0.001 vs. DM without erianin. (**H**) The expression of Iba1 in retinas. The representative pictures of retinal immunofluorescence staining of Iba1 and DAPI, and also the Merge of Iba1- and DAPI-stained images were shown at the left (Scale bars: 20 μm). The enlarged representative pictures of retinal Iba1-stained images were shown at the right (Scale bars: 5 μm). Red arrows indicate microglial cells.

Staining with CD31 is generally used to indicate the presence of vessels. Fig. 7A is the picture of the whole mounting of CD31-stained retinas, while Fig. 7B is the partial enlarged picture of CD31-stained retinas. From Fig. 7B, we can see that there were more CD31-stained vessels in STZ-induced hyperglycemic mice (Fig. 7B-b) than in normal control mice (Fig. 7B-a), whereas erianin (1, 10 mg/kg) reduced the increased retinal vessels in STZ-induced hyperglycemic mice (Fig. 7B-c,d). After counting the number of retinal vessels (Fig. 7C), we found that retinal vessels were increased in STZ-induced hyperglycemic mice, but erianin (1, 10 mg/kg) reduced such increase. Figure 7D showed the morphological changes of retinas in mice. There were more vessels appeared in GCL (ganglion cell layer), INL (inner nuclear layer) and OPL (outer nuclear layer) in STZ-induced hyperglycemic mice than in normal control mice (Fig. 7D-a,b). After erianin (1, 10 mg/kg) treatment, the increased vessels were reduced (Fig. 7D-c,d). Next results showed that VEGF content in serum and vitreous cavity were both obviously increased in STZ-induced hyperglycemic mice, however erianin (1, 10 mg/kg) reduced the increased VEGF content in both serum and vitreous cavity (Fig. 7E,F). In addition, erianin (1, 10 mg/kg) also reduced the increased mRNA expression of VEGF in retinas in STZ-induced hyperglycemic mice (Fig. 7G). However, erianin had no effect on blood glucose concentration and body weight in STZ-induced hyperglycemic mice (Supplementary Fig. 5). Ionized calcium-binding adapter molecule 1 (Iba1) is an often used biomarker for microglia^21^. Next, we observed the expression of Iba1 in retinas from STZ-induced hyperglycemic mice by using Iba1 immunofluorescence. As shown in Fig. 7H, the number of Iba1-positive microglia was increased in OPL, the inner plexiform layer (IPL) and GCL in STZ-induced hyperglycemic mice than in normal control mice. Moreover, such increase was reduced in erianin (1, 10 mg/kg)-treated mice.

### Erianin attenuated retinal neovascularization in OIR mice

**Figure 8:**
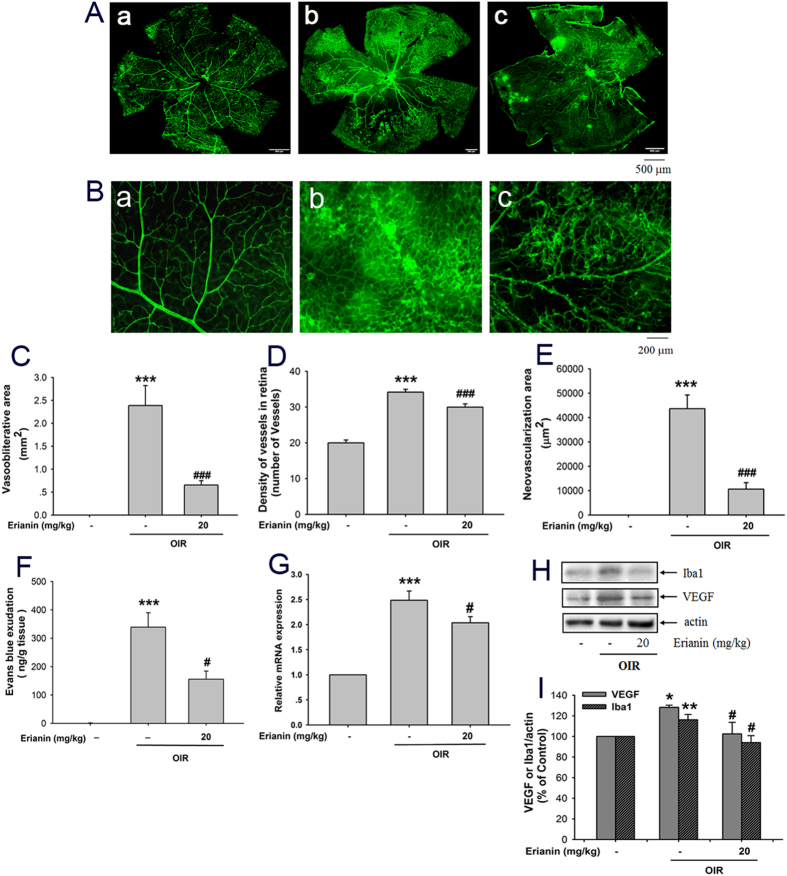
Effects of erianin on ischemia-induced retinal neovascularization. Mice were exposed to 75% oxygen from P7 to P12, and then returned to room air and received an intraperitoneal injection of 20 mg/kg/day of erianin for 5 days. (**A**) The whole mounting of CD31-stained retinas. (**B**) The partial enlarged picture of CD31-stained retinas at the peripheral area of retinas. (**C**) Calculating the vasoobliterative area at the central area of retina from OIR mice (n = 6). (**D**) Quantitative results of CD31-stained vessels at the peripheral area of retina from OIR mice (n = 6). (**E**) The neovascularization area based on the formed capillary hemangioma at the peripheral area of retina from OIR mice was calculated (n = 6). (**F**) Retinal vascular leakage was detected by using Evans blue leakage assay (n = 6). (**G**) The mRNA expression of retinal VEGF in OIR mice (n = 7). (**H**) Erianin reduced retinal VEGF and Iba1 protein expression in OIR mice. Results represent four repeated experiments. (**I**) Quantitative densitometric analysis of VEGF, Iba1 and actin, and the results were presented as percentage of control (n = 8). Data = Means ± SEM. *P < 0.05, **P < 0.01, ***P < 0.001 vs. control; ^*#*^*P* < 0.05, ^*###*^*P* < 0.001 vs. OIR without erianin.

The above results implied that erianin-induced alterations in retinal vessels can be achieved without a reduction of hyperglycemia in STZ-induced hyperglycemic mice. To further confirm whether the amelioration of erianin on DR occurred via the direct effects on the retina but was not due to the reduced blood glucose, nondiabetic OIR mice were used, which is a model of ischemia-induced retinopathy. Erainin was administered intraperitoneally once a day for 5 days from P12 in OIR mice. Figure 8A is the picture of the whole mounting of CD31-stained retinas at P17, while Fig. 8B is the partial enlarged picture of CD31-stained retinas. The results showed that erianin (20 mg/kg) reduced the area of vascular obliteration at the central retina and attenuated retinal neovascularization at the peripheral area of the whole retina (Fig. 8A,B). After calculating the vasoobliterative area at the central area of retina, we found that erianin (20 mg/kg) reduced the increased vasoobliterative area in retinas from OIR mice (Fig. 8C). After counting the number of vessels and calculating the neovascularization area based on the formed capillary hemangioma at the peripheral area of retina, we found that the number of vessels and the neovascularization area were both increased in OIR mice, but erianin (20 mg/kg) reduced such increase (Fig. 8D,E). In addition, the retinal vascular permeability assay showed that erianin (20 mg/kg) reduced retinal vascular leakage as compared with vehicle-treated OIR mice (Fig. 8F). Further results showed that erianin (20 mg/kg) reduced the increased mRNA (Fig. 8G) and protein expression of VEGF (Fig. 8H,I) in retinas from OIR mice. As shown in Fig. 8H,I, erianin (20 mg/kg) also reduced the protein expression of Iba1 in retinas from OIR mice.

## Discussion

A typical feature of diabetes is hyperglycemia, which will cause damage to endothelial cells and induce angiogenesis through promoting the expression of a variety of pro-angiogenic factors including VEGF (also known as VEGFA)^19^,^20^,^22^. Thus, high content of blood glucose is considered to play an important role in regulating the development of DR. In this study, retinal endothelial RF/6A cells were used to imitate the pathogenesis of DR under the condition of high content of glucose. Similar to other previous reports, our results showed that HG induced angiogenic responses in RF/6A cells including tube formation and cell migration^23^,^24^. However, erianin blocked HG-induced tube formation and migration in RF/6A cells in the concentration-dependent manner (Fig. 1). In addition, erianin also reduced the increased retinal vessels in STZ-induced hyperglycemic mice (Fig. 7A–D). All these results evidenced the abrogation of erianin on retinal angiogenesis induced by hyperglycemia during the process of diabetes. Moreover, erianin also reduced the increased number of vessels and neovascularization area in the peripheral area of retinas and attenuated retinal vascular leakage in OIR mice (Fig. 8A–F). These results implied that the inhibition of erianin on retinal neoangiogenesis was not dependent on the reduction of blood glucose.

A variety of reports demonstrated that VEGF content was elevated in serum or vitreous cavity prior to the appearance of discernible vascular lesions, and there were positive correlations between VEGF and blood glucose levels^25^,^26^,^27^. The source of elevated VEGF content in DR is still not fully elucidated, but may include the autocrine mechanism in retinal endothelial cells^23^,^24^,^28^, and the paracrine mechanisms in retinal pigment epithelial (RPE) cells or macroglia cells^29^,^30^,^31^. Endothelial cell-derived VEGF has recently shown to be an essential autocrine mechanism for the increased angiogenic activity in endothelial cells^32^. Microglial cells have been reported to be activated and play important roles in regulating retinal neuroinflammation during the development of DR^33^,^34^. However, whether there is microglia-derived paracrine VEGF mechanism under hyperglycemic condition remains unknown. HIF-1α is reported to be a well-known transcription factor for regulating the expression of VEGF^35^. In this study, we found that erianin reduced the HG-induced increased VEGF mRNA and protein expression, and also abrogated HG-induced HIF-1α transcriptional activation in both retinal endothelial RF/6A cells (Fig. 2) and microglia BV-2 cells (Fig. 3) *in vitro*. Erianin also reduced the elevated VEGF contents in both serum and vitreous cavity in STZ-induced hyperglycemic mice *in vivo* (Fig. 7E,F). In addition, erianin further reduced the increased retinal VEGF expression in STZ-induced hyperglycemic mice (Fig. 7G) and OIR mice (Fig. 8G,I). Furthermore, erianin also reduced the increased microglia activation in STZ-induced hyperglycemic mice (Fig. 7H) and OIR mice (Fig. 8H,I). All these above results evidenced that erianin reduced the increased VEGF expression during the development of DR, and such phenomena is related with its inhibition on HIF-1α-mediated autocrine and paracrine VEGF expression in both endothelial and microglial cells.

ERK1/2 is a subfamily member of MAPKs, which plays a critical role in regulating cell survival, proliferation and differentiation^36^. Previous reports have demonstrated that ERK1/2 played critical roles in regulating HIF-1α activation and VEGF expression during tumor development^37^,^38^,^39^. ERK1/2 was not required for hypoxia-induced HIF-1α and VEGF expression in RPE cells^40^. However, whether ERK1/2 will play critical roles in regulating HG-induced HIF-1α activation and VEGF expression in retinal endothelial cells or microglial cells remains unknown. Our present study showed that erianin inhibited HG-induced ERK1/2 phosphorylation in both RF/6A and BV-2 cells just like U0126 (Fig. 4A,B,E,F), which is a well-known MEK1/2 inhibitor and can effectively block ERK1/2 activation. Furthermore, U0126 and erainin both abrogated HG-induced HIF-1α transcriptional activation in RF/6A and BV-2 cells (Figs 2D,E, 3D,E and 4C,D,G,H). The above results indicate that erianin-induced inhibition on ERK1/2 activation contributes to its blockage on HIF-1α transcriptional activation and subsequent VEGF expression in retinal endothelial and microglial cells.

Further, the inhibition of erianin on VEGF-induced angiogenesis *in vitro* and *in vivo* was observed. The results showed that erianin abrogated VEGF-induced cell growth, migration and tube formation in retinal endothelial cells *in vitro*, and inhibited VEGF-induced neoangiogenesis in matrigels *in vivo* (Fig. 5). In addition, erianin alone had no obvious cytotoxicity on RF/6A cells (Fig. 1E). VEGF binds and activates two tyrosine kinase receptors: VEGFR1 and VEGFR2, of which VEGFR2 plays critical roles in the development of various pathological angiogenesis including DR by regulating proliferation and migration of retinal endothelial cells through distinct signal pathways^22^,^41^. Both cRaf-MEK1/2-ERK1/2 and PI3K-AKT-mTOR-P70S6kinase are the downstream signaling cascades of VEGFR2, and play critical roles in regulating VEGF-induced angiogenic responses in endothelial cells^42^,^43^. Our results demonstrated that erianin blocked VEGFR2 phosphorylation induced by VEGF; and it also abrogated the VEGF-induced activation of c-Raf-MEK1/2-ERK1/2 and PI3K-AKT-mTOR-P70S6kinase signaling cascades in retinal endothelial RF/6A cells (Fig. 6). All those results demonstrated that erianin blocked the activation of VEGF-stimulated VEGFR2 and its downstream c-Raf-MEK1/2-ERK1/2 and PI3K-AKT signaling cascades, which will contribute to its anti-angiogenic activity *in vivo* and *in vitro*.

In summary, our results demonstrate that natural compound erianin inhibits HG-induced retinal angiogenesis by blocking HG-induced VEGF expression through ERK1/2-mediated HIF-1α transcriptional activation in both retinal endothelial and microglial cells, and further abrogates VEGF-induced retinal angiogenesis by blocking the activation of VEGFR2 and its downstream cRaf-MEK1/2-ERK1/2 and PI3K-AKT signaling cascades. The present study indicates the potential application of erianin for DR treatment, and also provides an explanation for the eye-improving capacity of medicinal *Dendrobium* (Shi-Hu) for centuries.

## Methods

### Materials

Erianin was purchased from Shanghai Yuanye Bio-Technology Co., Ltd, and its purity is above 98.5%. Antibodies for VEGFR2, phospho-VEGFR2 (Tyr1175), c-Raf, phospho-c-Raf (Ser338), MEK1/2, phospho-MEK1/2 (Ser221), ERK1/2, phospho-ERK1/2 (Thr202/Tyr204), p38, phospho-p38 (Thr180/Tyr182), PI3K, phospho-PI3K p85 (Tyr458), AKT, phospho-AKT (Thr308), mTOR, phospho-mTOR (Ser2448), phospho-P70S6kinase (Thr389), HIF-1α, actin, and lamin B1 were all obtained from Cell Signaling Technology. Antibody for VEGF and ImmunoCruz goat ABC staining system were bought from Santa Cruz Biotechnology. All secondary peroxidase-conjugated antibodies were obtained from Jackson ImmunoResearch Laboratories. Cluster of differentiation 31 (CD31) antibody, fluorescein isothiocyanate (FITC) conjugated anti-Rat IgG and Alexa Fluor 488 goat anti-Rabbit IgG were purchased from BD Biosciences. Antibody for Iba1 was purchased from GeneTax, Inc. Human recombinant VEGF (isoform 165) was purchased from PeproTech. Cell culture reagents, Trizol, Matrigel, and 4′,6-Diamidino-2-phenylindole (DAPI) were purchased from Life Technology. RNeasy Plus Mini kit was purchased from Qiagen. Whole cell protein extraction kit and enhanced chemiluminescence kit were obtained from Millipore. PrimeScript RT Master Mix and SYBR Premix Ex Taq kits were purchased from Takara. NE-PER nuclear and cytoplasmic extraction kit was purchased from Thermo Scientific. U0126 were purchased from Alexis Biochemicals. D-Glucose and other reagents were from Sigma Chemical Co.

### Cell culture

The monkey choroid-retinal endothelial cell line RF/6A (ATCC, Manassas, VA) and microglia cell line BV-2 (ATCC) were cultured in Roswell Park Memorial Institute 1640 medium (RPMI1640) and Dulbecco’s modified eagle medium (DMEM) (Invitrogen, Carlsbad, CA) supplemented with 10% fetal bovine serum (Invitrogen), 100 units/ml penicillin and 100 μg/ml streptomycin (Invitrogen). Cells were incubated in a humidified atmosphere of 5% CO2 at 37 °C.

### Experimental animals

C57BL/6 male mice (8-week-old) were purchased from Shanghai Laboratory Animal Center of Chinese Academy of Sciences, and housed under specific pathogen-free conditions according to the guidelines of the association for Assessment and Accreditation of Laboratory Animal Care. All animals received humane care according to the institutional animal care guidelines approved by the Experimental Animal Ethical Committee of Shanghai University of Traditional Chinese Medicine, and the methods for *in vivo* study were carried out in accordance with the approved guidelines. All the experimental protocols were approved by the Experimental Animal Ethical Committee of Shanghai University of Traditional Chinese Medicine.

### Tube formation assay

RF/6A cells were starved in RPMI1640 containing 0.1 BSA% for 4 h, and then incubated with or without erianin for 15 min. Cells were seeded into 96-well plates pre-coated with Matrigel, and then treated with or without VEGF (10 ng/ml) or HG (25 mM) for another 4 h. Images were pictured under the inverted microscope, and tubes forming intact networks were counted in a blind manner.

### Cell migration assay

RF/6A cells were starved in RPMI1640 containing 0.1 BSA% for 4 h, and then incubated with or without erianin for 15 min. Cells were seeded into the upper chamber of the transwells pre-coated with gelatin, and then VEGF (10 ng/ml) or HG (25 mM) was added into the lower chamber. The non-migrated cells were wiped off with a cotton swab after 8 h, and the migrated cells were stained by crystal violet. Images were pictured under the inverted microscope, and the migrated cells were counted in a blind manner.

### Protein extraction

Nuclear and cytoplasmic proteins in cells were isolated as described in NE-PER nuclear and cytoplasmic extraction kit. Other cellular proteins were extracted by using a whole cell protein extraction kit. Retinas were homogenized in ice-cold lysis buffer containing 50 mM Tris, pH7.5, 150 mM NaCl, 1 mM EDTA, 20 mM NaF, 0.5% NP-40, 10% glycerol, 1 mM phenylmethylsulfonyl fluoride, 10 μg/ml aprotinin, 10 μg/ml leupeptin, and 10 μg/ml pepstatin A. After centrifugation, protein concentration of the resulting supernatants was determined. The protein concentration in each sample was normalized to the equal protein concentration.

### Western-blot analysis

Protein samples were separated by SDS-PAGE and blots were probed with an appropriate combination of primary and secondary antibodies. The antibody-reactive bands were identified by enhanced chemiluminescence kits. The grey densities of the protein bands were normalized using β-actin or lamin B1 density as an internal control, respectively.

### RNA isolation and cDNA synthesis

Cellular and retinal total RNA were isolated by using an RNeasy Plus Mini kit and Trizol reagents according to the manufacturer’s instructions. The RNA content was determined by measuring the optical density at 260 nm, and cDNA was synthesized by using PrimeScript RT Master Mix kit.

### Real-time PCR assay

Real-time PCR was performed by using SYBR Premix Ex Taq kit, and the relative expression of target genes was normalized to GAPDH in RF/6A cells or actin in BV-2 cells and in mice retinas, analyzed by the 2^−ΔΔCt^ method and given as ratio compared with the control. The primer sequences used in this study are shown in Supplementary Table.

### Cell viability assay

RF/6A cells (2 × 10^4 ^cells/well) were seeded into 96-well plates. After attachment, cells were pretreated with different concentrations of erianin for 15 min, and then incubated with or without VEGF (10 ng/ml) for 48 h. Cell viability was determined by 3-(4,5-dimethylthiazol-2-yl) 2,5-diphenyltetrazolium bromide (MTT). Optical density was measured at 570 nm with 630 nm as the reference and cell viability was normalized as the percentage of control.

### *In vivo* matrigel assay

Matrigel containing 10 ng/ml VEGF, heparin (30 U/ml) and erianin (10, 50 nM) in liquid form at 4 °C was injected into the abdominal subcutaneous tissue along the peritoneal mid-line of C57BL/6 mice (n = 6). After 7 days, the mice were sacrificed and the matrigel plugs were taken out and pictured. The matrigel plugs were also prepared for immunohistochemistry.

### Immunohistochemistry

Matrigel plugs were fixed in 4% paraformaldehyde and embedded in paraffin. The fixed sections (5 μm) were stained with CD31 and the whole experimental procedure was described in our previous published paper^15^.

### STZ-induced hyperglycemic mice

Thirty mice were administered intraperitoneally (i.p.) with 55 mg/kg STZ for 5 consecutive days, while the other ten mice were injected with physiological saline and served as control animals. The concentration of serum glucose was measured 7 days after the last injection; the glucose concentration of 28 mice was >16.5 mmol/L in this study, and those mice were considered as diabetic mice and randomly divided into 3 groups. At 3 months after the injection of STZ, the mice were administered intraperitoneally with erianin (1 or 10 mg/kg per day) for 2 consecutive months. All mice were sacrificed at 5 months after the injection of STZ.

### Retinal CD31 immunofluorescence staining

The procedure of retinal CD31 immunofluorescence staining was performed as described in our previous published papers^15^,^44^,^45^. Retinal vessels were pictured under the fluorescence microscopy and the quantity of vessels was counted as described previously^15^,^44^,^45^. The quantification of the vasoobliterative area at the central area of retina and the neovascularization area based on the formed capillary hemangioma at the peripheral area of retina from OIR mice was measured by using cellSens Dimension 1.6 (IX81, Olympus, Japan).

### Histopathological observation

Retinas isolated from mice were fixed in 4% paraformaldehyde solution and embedded in paraffin, and then sectioned (5 μm), stained with haematoxylin and eosin (H&E), and observed under the microscopy.

### ELISA assay

The whole blood and vitreous cavity suspended in PBS was centrifuged at 5000 g, 4 °C for 10 min. The serum and supernatant of vitreous cavity were collected for further ELISA assay according to the manufacturer’s instruction.

### Immunofluorescence staining of Iba1in retinas

Paraffin-embedded sections of retinas (5 μm) were deparaffinized in xylene, and rehydrated in an ethanol gradient with distilled water. After endogenous peroxidase activity was quenched, retinas were incubated with 5% bovine serum albumin to minimize nonspecific binding. After rinsing three times (5 min each), retinas were incubated with Iba1 antibody at 4 °C overnight, and further incubated with Alexa fluor 488 goat anti-rabbit IgG (H + L) antibody at room temperature for 1 h after rinsing three times again. After rinsing three times again, retinas were incubated with DAPI for 10 min. Images were captured under an inverted microscope (IX81, Olympus, Japan).

### OIR mice

On postnatal day 12 (P12), after the C57BL/6 mice were exposed to 75 ± 2% oxygen for 5 days (P7-P12), the mice were returned to the normal room air and randomly divided into two groups: vehicle-treated OIR mice and erianin-treated (20 mg/kg/day) OIR mice. The normal control mice were kept under normal room conditions from birth until postnatal day 17 (P17). The mice were injected intraperitoneally with erianin (20 mg/kg per day) for 5 days. On P17, the mice were anesthetized and sacrificed.

### Measurement of retinal vascular leakage using Evans Blue Assay

Mice were injected with 2% Evans blue (10 μl/g, i.p.) in PBS. After 2 h, blood was extracted through the left ventricle; and the mice were perfused with PBS to completely remove the Evans blue dye in blood vessels. Retinas were carefully dissected and the weight was determined after thoroughly drying. Next, the retinas were incubated in 120 μl formamide for 18 h at 70 °C to extract the Evans blue dye. The extract was centrifuged at 10,000 g twice for 1 h at 4 °C. Absorbance of the supernatant was measured with a spectrophotometer at 620 nm. The concentration of Evans blue dye in extracts was calculated using a standard curve of Evans blue in formamide and normalized to the dried retinal weight.

### Statistical analysis

Data were expressed as means ± standard error of the mean (SEM). The significance of differences between groups was evaluated by one-way ANOVA with LSD post hoc test; and *P* < 0.05 was considered as indicating statistically significant differences.

## Additional Information

**How to cite this article**: Yu, Z. *et al*. Erianin inhibits high glucose-induced retinal angiogenesis via blocking ERK1/2-regulated HIF-1α-VEGF/VEGFR2 signaling pathway. *Sci. Rep.* **6**, 34306; doi: 10.1038/srep34306 (2016).

## Supplementary Material

Supplementary Information
