# Supplementary material for: Erianin inhibits high glucose-induced retinal angiogenesis via blocking ERK1/2-regulated HIF-1α-VEGF/VEGFR2 signaling pathway
Source: Sci Rep. 2016 Sep 28;6:34306. doi: 10.1038/srep34306 (PMC5039671; doi:10.1038/srep34306)
Supplement: Supplementary Information [file srep34306-s1.pdf]

Manuscript title: Erianin inhibits high glucose-induced retinal angiogenesis via blocking ERK1/2-regulated HIF-1α-VEGF/VEGFR2 signaling pathway

Author List: Zengyang Yu, Tianyu Zhang, Chenyuan Gong, Yuchen Sheng, Bin Lu, Lingyu Zhang, Lili Ji, Zhengtao Wang

Supplementary Table: List of Primers for Real-time PCR

Target	Gene ID	Primer	Sequence
VEGFA	574209	FP	5’-GCGGAATTCATCATGCGGATCAAACCTCACCA-3’
		RP	5’-TACGGATCCTCCGGACCCAAAGTGCTC-3’
GAPDH	574354	FP	5’-ACCACAGTCCATGCCATCAC-3’
		RP	5’-TCCACCACCCTGTTGCTGTA-3’
Vegfa	22339	FP	5’-GCTACTGCCGTCCGATTGAG-3’
		RP	5’-ACTCCAGGGCTTCATCGTTACAG-3’
Actb	11461	FP	5'-TTCGTTGCCGGTCCACACCC-3'
		RP	5'-GCTTTGCACATGCCGGAGCC-3'

FP, Forward Primer; RP, Reverse Primer

Manuscript title: Erianin inhibits high glucose-induced retinal angiogenesis via blocking ERK1/2-regulated HIF-1α-VEGF/VEGFR2 signaling pathway

Author list: Zengyang Yu, Tianyu Zhang, Chenyuan Gong, Yuchen Sheng, Bin Lu, Lingyu Zhou, Lili Ji, Zhengtao Wang

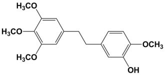

Supplementary Figure 1

The chemical structure of Erianin

Manuscript title: Erianin inhibits high glucose-induced retinal angiogenesis via blocking ERK1/2-regulated HIF-1α-VEGF/VEGFR2 signaling pathway

Author list: Zengyang Yu, Tianyu Zhang, Chenyuan Gong, Yuchen Sheng, Bin Lu, Lingyu Zhou, Lili Ji, Zhengtao Wang

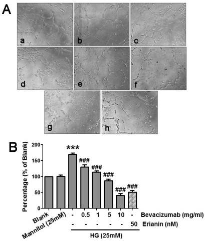

Supplementary Figure 2

Bevacizumab inhibited HG-induced tube formation in RF/6A cells. (A) Cells were pretreated with different concentrations of bevacizumab and erianin (50 nM) for 15 min, and then incubated with HG (25 mM) for 4 h. The formed tube-like structures were taken under microscope. a. Blank, b. Mannitol (25 mM), c. HG, d. HG+ bevacizumab (0.5 mg/ml), e. HG+ bevacizumab (1 mg/ml), f. HG+ bevacizumab (5 mg/ml), g. HG+ bevacizumab (10 mg/ml), h. HG+ erianin (50 nM). (B) Tube-like structures were quantified by manual counting, and presented as percentage of Blank (n=3). Data= Means ± SEM. ***$P<0.001$ vs. Blank; $^{###}P<0.001$ vs. HG.

Manuscript title: Erianin inhibits high glucose-induced retinal angiogenesis via blocking ERK1/2-regulated HIF-1α-VEGF/VEGFR2 signaling pathway

Author list: Zengyang Yu, Tianyu Zhang, Chenyuan Gong, Yuchen Sheng, Bin Lu, Lingyu Zhou, Lili Ji, Zhengtao Wang

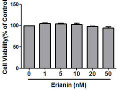

Supplementary Figure 3

Effects of Erianin on cell viability in BV-2 cells. BV-2 cells were incubated with different concentrations of erianin for 12 h, and then cell viability was detected by MTT assay (n=6). Data= Means ± SEM.

Manuscript title: Erianin inhibits high glucose-induced retinal angiogenesis via blocking ERK1/2-regulated HIF-1α-VEGF/VEGFR2 signaling pathway

Author list: Zengyang Yu, Tianyu Zhang, Chenyuan Gong, Yuchen Sheng, Bin Lu, Lingyu Zhou, Lili Ji, Zhengtao Wang

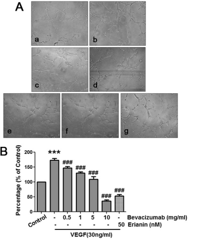

Supplementary Figure 4

Bevacizumab inhibited VEGF-induced tube formation in RF/6A cells. (A) Cells were pretreated with different concentrations of bevacizumab and erianin (50 nM) for 15 min, and then incubated with VEGF (30 ng/ml) for 4 h. The formed tube-like structures were taken under microscope. a. Control, b. VEGF, c. VEGF+ bevacizumab (0.5 mg/ml), d. VEGF+ bevacizumab (1 mg/ml), e. VEGF+ bevacizumab (5 mg/ml), f. VEGF+ bevacizumab (10 mg/ml), g. VEGF+ erianin (50 nM). (B) Tube-like structures were quantified by manual counting, and presented as percentage of Control (n=3). Data= Means ± SEM. ***$P<0.001$ vs. Control; ###$P<0.001$ vs. VEGF.

Manuscript title: Erianin inhibits high glucose-induced retinal angiogenesis via blocking ERK1/2-regulated HIF-1α-VEGF/VEGFR2 signaling pathway

Author list: Zengyang Yu, Tianyu Zhang, Chenyuan Gong, Yuchen Sheng, Bin Lu, Lingyu Zhou, Lili Ji, Zhengtao Wang

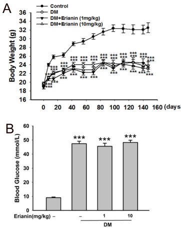

Supplementary Figure 5

A. Body weight, B. Blood glucose content
